# The First Year Developmental Crisis: Origin of Cultural Action

**DOI:** 10.3389/fpsyg.2021.686761

**Published:** 2021-08-20

**Authors:** Yulia Solovieva, Luis Quintanar

**Affiliations:** ^1^Meritorious Autonomous University of Puebla, Puebla, Mexico; ^2^Autonomous University of Tlaxcala, Tlaxcala, Mexico

**Keywords:** early childhood, developmental crisis, Vigotsky’s conception of development, developmental assessment, guiding activity of early age

## Abstract

According to the conception of cultural historical psychology, introduced by L. S. Vigotsky, the first year of a child’s life represents a specific period of development or the first psychological age. Psychological development should be differentiated from biological development and have proper objective indicators. Psychological development starts with the possibility of initial cultural communication between an adult and child, within a unique kind of social situation of development. The goal of the article is to describe the content of the crisis of the first year of life as a psychological phenomenon and to propose psychological and neuropsychological indicators for qualitative assessment of the progress of psychological development at the end of the first year of life. The article opens the discussion about guiding the activity of the first age, new psychological formations of the crisis of the first year, and qualitative changes in the social situation of development. The content of the first psychological age and crisis is presented in the article, according to proposals of cultural historical psychology. Future interdisciplinary research should be continued in order to establish optimal strategies for adult and child interaction during a stable period of development and the crisis of the first year.

*“Relation between the child and reality, from the very beginning, is social relation. In his sense, the human newborn is the social being by excellency [sic].”*

L. S. Vigotsky

## Introduction

The first year of life should be understood as a specific period of development or as a particular psychological age, including a stable period of development with specific phases and the critical period. This period has its own content and structure, quite different from the content of later psychological ages. Psychological age cannot be determined only by chronological limits, but has to be analyzed according to developmental manifestations of each child with normal and abnormal development (Vigotsky, 1984).

According to the conception of cultural development, introduced by L. S. Vigotsky in psychology, psychological development cannot be reduced to biological development, physiological processes, or neurological maturation (Leontiev, 2009; Lisina, 2009). Psychological age is a period of ontogenetic development characterized by proper manifestation of a child’s activity and personality. Central and accessory lines of development exist within the guiding activity of the age. Psychological age includes specific guiding activity, which leads to gradual acquisition of new psychological formations (Elkonin, 1995). New psychological formations might be acquired as a result of constant joint activity between adult and child. Such joint activity occurs in conditions of specific social situations of development. New psychological formations appear at a critical period and reflect a developmental crisis. New psychological formations are qualitative characteristics of the child’s activity and personality, which appear at the end of the age and announce the critical period, as the passage to the next period of age (Vigotsky, 1984; Obukhova, 2006). According to Bozhovich (1981, pp. 130, 131), “new psychological formation appears as a specific sequence, which characterize[s] the periods of central line of their ontogenetic development.” We suppose that the study of this sequence of new psychological formation in infancy has not yet been completed.

In each case of psychological development, a social situation of development might be negative or positive in relation to the guiding activity of the child. Social situations of development might be understood as the actions of the adult directed to the child during each period of development. These actions might be favorable or unfavorable for guiding the activity of psychological age (Solovieva and Quintanar, 2020).

Vigotsky (1984) has written that the problem of psychological age is the central problem of psychological research and of psychological assessment. The task of developmental psychology is to study the content of each psychological age in positive and negative social and organic conditions of development.

Psychological development starts with the possibility of initial cultural communication between an adult and child, within a unique kind of social situation of development. According to cultural historical perspective in psychology, an adult is a representative of cultural experience, which has to pass from them to the child (Obukhova, 2006; Ilienkov, 2009). The child’s activity is not an individual activity from the very beginning, but is an act of communicative collaboration with an adult. The adult’s activity, directed to the child, is the unique origin of a child’s cultural development.

Each psychological age might be studied and assessed according to the terms of social situations of development, guiding activity, new psychological formations, central and accessory lines of development, and the crisis of the age as the bridge for the next period of development. Table 1 shows the structure of each psychological age.

**TABLE 1:** The structure of psychological age.

Element of the structure of psychological age	Description	Indicators/agents
Social situation of development	Type of relations between the child and society in each age	Actions and attitudes of adults and institution in relation to the child
Guiding activity of the age	Psychological activity, which leads to development in this age	Motivated activity of the child; interests and intentions of the child
Central line of development	Achievements of development during the age	Affective or practical experience of the child as new manifestations of development
Accessory line of development	Achievement of previous age or conformation of the basis for the next age	Affective or practical experience of the child as basis of development
New psychological formations	New qualitative features of activity and personality at the end of the age	Concrete indicators of development, which might be discovered by qualitative assessment
Stable phases	Long period of development within the same social situation	Observation of social situation of development and guiding activity
Critical phase	Brief manifestation of the need for changing of social situation of development	Psychological assessment of new formations of the age with observation of social situation of development and guiding activity

The concrete content of psychological and neuropsychological assessment of stable and critical periods of development is one of the essential problems in developmental psychology in cases of children with optimal development and with difficulties (Solovieva and Quintanar, 2016).

The goal of the article is to describe the content of the crisis of the first year of life as a psychological phenomenon and to propose psychological and neuropsychological indicators for qualitative assessment of progress of psychological development at the end of the first year of life. The main concepts introduced by Vigotsky (1984) and his followers (Leontiev, 1984, 2003; Elkonin, 1980, 1995; Lisina, 2009) are used as theoretical background for precision of our ideas.

## The Content of the First Psychological Age

Let us revise the structure of the first psychological age. Guiding activity during this period might be understood as activity of communication between adult and child. Communication is not a natural expression of a child’s individual life, but a kind of cultural activity that has its own motives, goals, means, results, and operations (Lisina, 2009). This kind of activity has to be gradually formed from an external level of social communication into an internal level of the child’s activity. Bozhovich (1981) stresses that the consciousness of the baby at the beginning of the first year of life represents mostly emotional components, related to immediate interactions.

How does this communication start, as it is obvious that the child is born without any kind of cultural communication and even language would appear as the result of development at the end of the first year of life, in cases of possible development? How is it possible to convert some kinds of diffuse immediate interactions, related to the emotions of the child, into meaningful process of one’s own cultural activity?

Cultural communication starts with the clear expression of communicative intention. Commonly, it is possible to notice this intention between the end of the first month and the third month of a child’s life. From the age of five months, such complex communication, with notable expression of a child’s smile directed to an adult’s face, might be consolidated (Bazhenova et al., 2007).

Initial intention for communication, represented as a complex of animation, includes four essential elements, which might be identified in the situation of communication between adult and child. We propose to assess these elements as follows:

1 Exchanging of mutual eye contact between adult and child.
2 General non-specific corporal agitation and movements of arms and legs of the child in direction to the adult.
3 Smile of the child in response to the smile of the adult.
4 Non-specific vocal expression of the child in response to the actions and speech of the adult.

**FIGURE 1:**
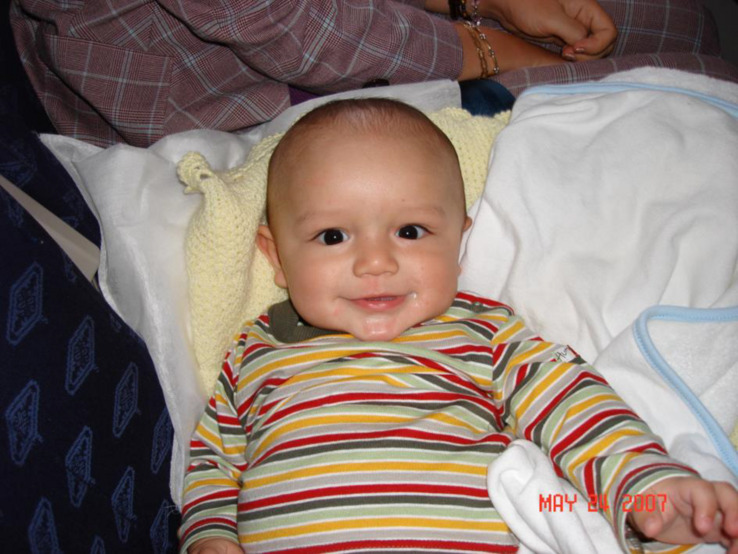
Complex of animation during the first months of life.^1^

All these four elements should appear simultaneously and in the presence of the adult, together with the whole situation of joint communication with the adult. Isolated movements, such as sounds or even a smile as reaction to physiological satisfaction, are not indicators of animation complex. The presence of the mentioned components of animation complex might be identified only in the situation of adult and child communication, so that psychological assessment in this age should be conducted only in the presence of the close adult in the situation of close affective communication. Figure 1 presents a clear example of such a situation.

All these elements conform to the famous animation complex, introduced in literature by Lisina (1986, 2009). The animation complex can be defined as an indicator of the start of a child’s communication activity with proper intention; this intention becomes conscious at the end of the first year of life. The periods of duration of animation complex may be several minutes long and is expressed mainly between the second and eight months of a child’s life. After that, the animation complex disappears which opens the path for changing of means of communication between adult and child and is characterized by the inclusion of objects and the first manifestation of words or of “autonomous speech” in terms of Vigotsky (1982).

The concept of animation complex is essential for identification of the start of communicative activity as the child’s own activity (Lisina, 2009). With communicative expression, the child converts into the subject of proper activity. Vigotsky has written that the newborn child is the most social being and that the whole process of development should be understood as the process of “individualization” of the child’s psyque. Such a position is contrary to the traditional way of understanding development as the process of “socialization” (Piaget, 1976). Different lines of psychological development were proposed by prominent psychologists of the last century: “individualization” (Vitgotsky) and “socialization” (Piaget, 1976). It is possible to refer to these positions as two general paradigms: a paradigm of cultural historical development, based on proper activity of psychological subject, and a paradigm of constructivism, based on biological maturation as the feature of natural evolution (Solovieva and Quintanar, 2019).

Communicative activity is the only kind of activity that provides positive appropriation of general cultural experience during the first year. Absence of this activity may have negative consequences in psychological development, such as the absence of appearance of language at the critical period, absence of actions with objects, and absence of independent movements directed to goals.

It is very important to stress that communication would never start automatically from the child, but is a result of an adult’s efforts to provide social joint communication. According to Vigotsky (1984), psychological functions of the child are initially divided between an adult and a child. That is why the child’s functions are social from the very beginning and become individual only later.

It is important to remember that there is no communicative activity of the child without communicative activity with an adult. At the same time, this activity should be positive affective communication. The indifference of an adult evokes the indifference of the child and their negative attitude provokes a negative response from the child.

Table 2 shows the content of the first psychological age according to general structure of the age.

**TABLE 2:** The content of the first psychological age.

Element of the structure of psychological age	Description	Indicators/agents
Social situation of development	All kinds of situations of close emotional contact between adult and child	Concrete actions and attitudes of adults, which guarantee close affective communication in day-to-day life
Guiding activity of the age	Affective emotional communication with an adult	Interest in an adult and aspiration for contact
Central line of development	Affective emotional positive basis for personality	Concrete affective episodes of communication
Accessory line of development	Practical experience of manipulation with objects and toys	Concrete practical episodes with objects
New psychological formations at the end of the age	Locomotion Autonomous speech Actions with objects	Aspiration to stand up and make passes; intentions of articulation of words; intentions for usage of objects independently
Stable phases	The first year of life as the period of emotional communication with two phases	Phase of communication without objects and with objects, starting from the second half of the first year
Critical phase	Manifestation of independency development	Aspiration of separation from adults for more independent movements and actions; appearance of new age formations

## Communicative Activity and Its Brain Organization in the First Year of Life

The adult organizes the life of the child; the adult decides when to move, to carry, to feed, to bathe, to go to sleep, when to go for a walk, or when to play. The adult establishes the goals of the child’s communication, provides different objects, and offers the verbal determination of objects and situations. The conformation of functional relations between unions of nervous mechanisms might be organized only as a result of cultural activity directed to previously established goals. The human brain is not the source of psychological functions, but functional level of realization of human cultural activity, which conforms to functional systems (Anokhin, 1980) or functional organs during a child’s active life (Leontiev, 2000). Functional systems in humans are not only the result of natural maturation, but also of cultural activities (Bernstein, 2003; Machinskaya, 2012), which emerge as joint activities guided by an adult.

Parameters of the structure of psychological age may serve as a proposal of qualitative assessment of psychological development of the child. The content of the guiding activity might be used for study and assessment of brain mechanisms, which take part in this activity.

Communication as a kind of human activity cannot be based on only one isolated “area” of development or only one brain zone or one neural net (Gazzaniga, 1993; Bassett and Gazzaniga, 2011; Dehaene, 2015). Each cultural activity requires functional organization and participation of the whole central nervous system, including three functional units: unit of general brain activation, unit of processing of sensorial external and internal information, and unit of programming and control (Luria, 1969). Developmental neuropsychology intends to discover and follow the formation of such “factors” through diverse periods of ontogenetic development (Solovieva and Quintanar, 2016). The first period of ontogenetic development is the age of joint emotional affective communication between adult and child (Solovieva et al., 2016).

The structure of joint communicative activity includes the social need expressed in concrete motives: an adult, who organized a child’s communication. The means or operations of this communication are corporal movements, eye movements, vocalization, and facial expressions of the child. All these means require participation of different levels of hierarchic brain organization, including cortical, subcortical, and cortico-subcortical relations. In this sense, it is not useful to speak about isolated areas of motor development, personality development, and speech development, as communication involves movements, expressions, and vocalizations. The absence of communication results in severe stagnation in a child’s psychological development not only during the first year, but also during further periods of development.

Communicative activity between the adult and child starts with the animation complex. The appearance of this complex can serve as an indicator of appearance of a new functional system, which includes diverse mechanisms of systemic and hierarchic brain organization, or three functional units, according to Luria’s (1973) theory.

Firstly, the animation complex requires general non-specific activation of the cortex; general activation includes necessary mechanisms of emotional activation as affective attraction and involvement of the child into the process of communication. All subcortical structures of the brainstem and limbic circuits take part in this general activation (Luria, 1973).

Secondly, the animation complex includes processes of perception of all modalities: auditive (voice of adult), visual (face of adult), and tactile (gentle touching of an adult). Such perception guarantees formation of complex polimodal images and retention of significant information. Posterior zones of the brain cortex take part in this perception.

Thirdly, the whole emotional complex of animation is directed to an adult, who is the motive of this communication activity. The movements of the child’s eye and contact with the adult’s gaze is the central mechanism of this complex. In this case, the unit of programming and regulation of movements takes part, so that the eyes of the child might concentrate on the face of an adult for longer periods. Frontal lobes with zones of regulation of eye movements, but also of all level of programming of movements, guarantee this process. Essential participation of the anterior frontal (orbital) regions guarantees connections with the limbic circuit and thalamic system, which also provides relations of anterior cortex with all posterior brain zones and zones of processing of sensorial modalities (Bezrukikh and Farber, 2000; Bezrukikh et al., 2009).

This example shows that there is no reason to talk about each part of the brain or about each cognitive process in an isolated manner, for example, about motor reactions, perception, and speech as though they were independent isolated functions (Glozman, 2009; Akhutina and Pilayeva, 2012). Luria (1973) has expressed that it is a great mistake to think that each brain unit works separately for some kinds of cognitive functions, but that the general participation of all units guarantees human complex activity.

Actions of affective communication between adult and child provide visual sustained contact directed to a cultural goal. An important indicator of this sustained contact is the child’s smile, directed to the adult’s smiling face. It was precisely this child’s smile that was noticed by Vigotsky (1984) and studied in details by his followers (Lisina, 2009).

Functional brain organization starts together with animation of complex in the first months of the child’s life. The absence or late appearance of the animation complex also means the absence or late appearance of functional brain organization.

Communication permits to guarantee social necessity for new experiences and impressions, for example to share contact with others. According to Tomasello (2013), this necessity should be recognized as a basic human necessity. This social necessity is the main difference between development of a human child and superior apes, as was shown in different experiments (Tomasello, 2013). Tomasello (2013) proposes to refer to this necessity as empathy. We agree and believe that empathy is a result of early and proper organization of adult child system of communication, starting with the complex of animation.

Four elements of the content of the complex of animation might be clearly related to elements of functional brain organization. Table 3 proposes a relation between elements of the complex of animation (Lisina, 2009) and functional brain units, according to Luria’s (1973) conception.

**TABLE 3:** Animation complex and brain units.

Elements of animation complex	Description	Reference to brain functional organization
Eye’s contact	Concentration of the adult’s face and eyes	Frontal lobes, area of eye movements; posterior visual primary and secondary zones
Corporal agitation	Movements of arms and legs toward the adult	Motor primary and secondary zones, subcortical structures of organization of movements and muscle tone regulation
Smile	Response to the adult’s smile	Frontal lobes and limbic structures
Vocalization	Intents of articulation or emission of guttural sounds	Parietal and auditive sensory and secondary zones of both hemispheres

According to Table 3, it is possible to appreciate broad participation of the central nervous system in the animation complex. Very important is that all these brain mechanisms should appear to work simultaneously and not as isolated processes. These brain functional components, while supporting the animation complex, give origin to the unit of stable general brain activation, unit of sensorial perception, and unit of programming and control. The resting of any of these elements might result in disorganization of the whole functional system for positive affective communication with the adult with different possibilities of negative consequences on present and future psychological development.

At the same time, these four elements of the animation complex might serve as indicators of manifestation of directed activity of the child. It is possible to call them both indicators of psychological development and also of the first manifestation of conformation of the functional system of communication action from a neuropsychological point of view. The complex of animation is a positive indicator of first communication action and first functional system with its clear brain mechanisms.

These indicators depend only on actions and attitudes of an adult toward the child; later, the child shows more and more initiative and will direct his or her own attitudes and communicative actions toward other people and objects. Activity of positive affective communication will produce later important changes in the cognitive and affective “image of the world” of the child (Leontiev, 1984).

The zone of proximate development will turn into the zone of actual development for goals and expressions of communication. At the end of the first year, the child will be conscious of his or her communicative goals. In this case, the child would have conformed communicative actions directed to conscious goals of affective communication: to get, to show, to share, to possess, and so on. Communicative activity will be the basis for the next guiding activity: actions with cultural objects.

## Two Phases of Development of Communicative Activity During the First Year

Psychological development of the child during the first year suffers transformations; gradually, the “first sensorial generalizations appear, the baby starts to use elements of words and objects” (Bozhovich, 1981, p. 132). The central line of psychological development of affective representations starts to be accompanied by the accessory line of practical usage of the objects. Let us try to understand how it happens.

According to Lisina (2009), a child’s communicative activity possesses the following features, typical for this period of development:

1 Affective attention and interest of all actions of the adult.
2 Emotional response to each contact and action of an adult toward the child.
3 Initiative of the child to follow and to involve an adult in communication.
4 Sensibility of a child toward positive attitude of an adult, which he/she manifests to other persons, objects, and the child.

Detailed analysis of these characteristics of a child’s communicative activity during the first year of life allows to establish two different phases. Gradually, the child starts to pay attention not only to the adult’s face, but also to the objects and toys that the adult uses and shows to the child and names in oral speech. The absence and the presence of cultural objects with cultural meaning and the possibility of their usage in clear practical situations makes the difference between the phases of development of communication. According to Lisina (1986), two phases of development of communication between child and adult might be identified.

The first phase of psychological development during early age might be characterized by “communication in personal situations.” The first indicator of this activity is a complex of animation, based on exchanging of eye contact and long contact between adult and child, exchanges of smiles together with vocalizations, and general motor excitation of the child toward the appearance and communicative actions of an adult. Such communication is the first guiding activity of cultural development of the child. In cases of positive social situation of development, when an adult is able to guarantee and to start this communication, it starts at the end of the first or second month of a child’s life. The positive social situation of development implies constant communicative actions of the adult toward the child. Communication of the adult is always verbal and emotionally expressive, while the communication of the child is only emotionally expressive. All movements of the child are included in the content of this personal affective non-verbal communication with an adult. Within this activity, the adult presents and tries to put different attractive objects and toys in the child’s hand. The objects are not interesting for the child *per se*, but only as the objects possessed by an adult.

The cultural object and the possibility of touching and moving the objects opens new qualitative possibilities for the child’s development.

The second phase of the first year of life occurs as “practical communication” organized between adult and child. At this period, the child is already able to sit independently and can start to explore and manipulate the objects by himself/herself. The child starts to be fascinated by the appearance of new objects and starts to manipulate them. It even seems that the child is “obsessed” by objects (Lisina, 2009). The child needs constant help and participation of an adult in order to know what to do with different objects. The child also needs the adult to share affective communication and re-affirmation of their own practical intention. Affective communication is still the guiding activity of the age, but new means were already introduced in this activity. These means are cultural objects, offered and used by the adult and directed to the child. Practical initiative and orientation of the adult is an essential condition for further psychological development of each child in human society.

Affective communication in practical situations implies a variety of social situations for collaboration. Each adult should be conscious of the necessity of constant useful propositions of joint actions with the child. Suitable situations for these joint actions might be as follows: going for a walk, election and changing of clothes, admiration of flowers and animals, usage of a spoon, playing with a toy. The time that an adult spends with the child should be directed to the goals of positive affective communication and initiative for stating of practical usage of cultural objects of day-to-day life. It is essential to notice that first manipulations with objects starts before the child may speak and before the child may walk, so that manipulation with cultural objects is the second line of a child’s developmental, after the line of shared affective communication. Vigotsky (1982) was right when he wrote that emotions are alfa and omega of the whole process of development.

Cultural objects are not simply physical objects which have dimensions and sizes. Each cultural object is a product of the history of humanity and of constant production and usage of the objects (Obukhova, 2019). The social situation of development of the first year should consist of an adult’s actions, which guarantee both affective and practical involvement of the child into joint realization of communicative and simple practical actions. Alteration of this situation of development results in stagnation on a child’s development as absences of activity directed to the goals.

All forms of a child’s initiative should be approved by adults. Social initiative and positive approval are the basis for social motives of activity and the basis for the motive of cooperative activity and mutual help (Tomasello et al., 2005). Tomasello (2013) has affirmed that superior apes are not eager to help each other or another participant of an action, while 9-month-old babies with positive development show motivation for helping and cooperation. According to activity theory, motives of activity emerge as previously established and as shared goals of the same activity (Leontiev, 1984).

In the case of communication activity, the motives for communication might emerge as previously established goals of communication. For example, the adult might direct the attention of the child to an interesting object, such as the moon in the sky. In this case, the moon is a goal of the adult’s communication toward the child. Later on, the child would search for the moon in the sky and show it to the adult. In this case, the moon was the motive of communication of the child.

In order to provide meaningful relation of the child with the objects of the cultural world, the adult should introduce meaning to all joint actions. The goals of these actions will turn into conscious intentions and conscious goals of the child’s communication.

The content of the consciousness of the child for the end of the first year of life would include their own goals of positive communication. The crisis of the first year of life starts when the child starts to wish, unconsciously, to use and to possess independently more and more new interesting objects. The child even starts to show rejection of an adult’s help.

## The Content of the Crisis of the First Year

The crisis of development at the end of the first year is the result of the whole process of development of communicative activity in its two phases. This crisis serves as an indicator of positive qualitative change in a child’s activity and personality. The crisis is expressed by a child’s intentions for independent locomotion, articulation of first words, and the performance of actions with cultural objects and toys. This last point is the central new psychological formation of the first age of development. If manifestations of locomotion and articulation are well known in psychology, very little research is dedicated to identification and promotion of development of actions with objects (Solovieva et al., 2018, 2020).

The main feature of the content of the crisis is that the child starts to use the objects according to their cultural function and starts to do it well and independently.

This is the main new formation of the first psychological age and an expression of the crisis of the first year. In this case, the social situation of development should never be understood as a natural context of development, as is claimed in a majority of publications. We propose that the social situation of development might be understood technically as the actions of the adult toward the child at each period of development.

Cultural usage of objects should be differentiated from simple manipulation and exploration of objects and toys. Manipulation might be fulfilled separately by each hand, the child may move an object with no vocalization or without the presence and emotional support of an adult; there is no evidence of proper actions with an object, as the child may put it into their mouth, drop an object, just simply touch it without no usage, and so on. In the case of appearance of proper cultural actions, the main differential features are as follows:

1 Eye movements and concentration of the glance toward an object.
2 Usage of both hands in coordinated movements trying to achieve proper actions with an object.
3 Intention of articulation of the words for this object or action.
4 Sharing with an adult or direction of an action with an object toward the adult with aspiration of approvement or agreement of the adult.

**FIGURE 2:**
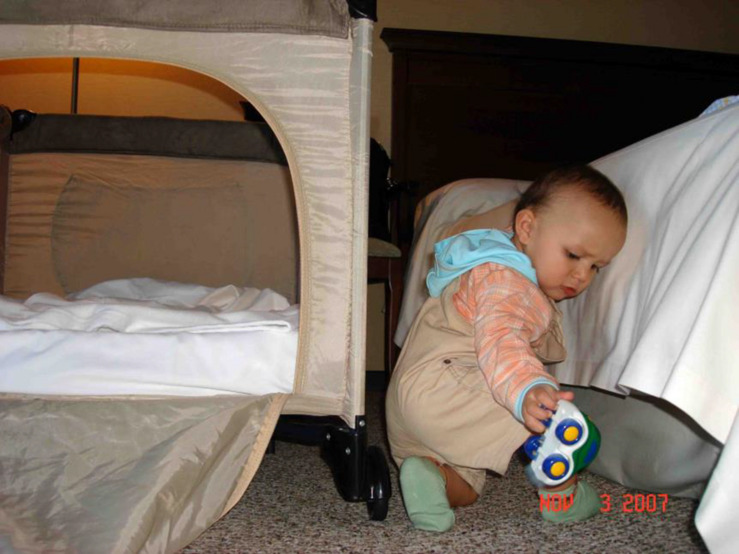
Manipulation with the toy (see text footnote 1).

**FIGURE 3:**
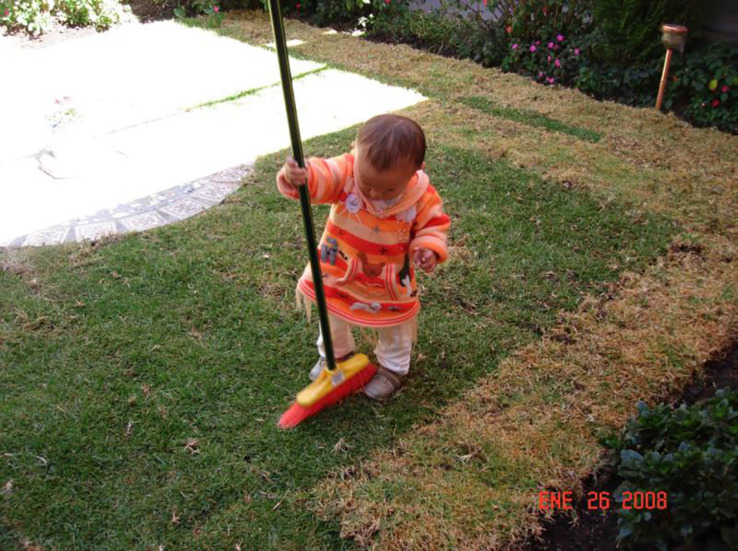
Action with the object (see text footnote 1).

The difference between manipulation and actions with cultural objects directed to the established goal is essential for proper organization of a child’s development during the crisis of the first year. Each adult has to give the child an opportunity to use a variety of cultural objects with the help, orientation, and oral explanation of the objects, actions, and situations. The adult continues to be representative of cultural experience and has to share this experience with the child. Figure 2 shows manipulation, while Figure 3 presents an example of the first cultural action in a child’s life.

On the basis of such affirmation, we may confirm that the child takes part in the act of cultural communication. According to Eco (2005), the act of cultural communication begins when the participant can recognize an object of his/her communication. We believe that such a position might be useful for understanding not only anthropological and semiotic situations, but also the beginning of communication at the early stages of development. In the case of cultural development, the adult names the object to the child together while showing the proper action with the object. Later on, the child may recognize the object together with the verbal name and the corresponding action. Each action is introduced to the child by an adult, but the child must fulfill the action actively with the help of an adult. According to Leontiev (2009), the child’s development is the acquisition of human actions, as a part of cultural history of humanity.

According to Table 4, it is possible to notice that the social situation of development might support, but also might be an obstacle for, the psychological development of the child. It is not enough to be with the child, but is also necessary to act in order to guarantee favorable psychological development. Assessment of the social situation of development might be helpful for elaboration of recommendations for parents and institutions for overcoming possible developmental difficulties (Solovieva and Quintanar, 2020). With the crisis of the first year, the next psychological period starts, in which systemic structure of the consciousness emerges; it happens while the meaning of historical and cultural objects becomes the meaning for the child (Vigotsky, 1984). Table 4 includes the psychological content of the crisis of the first year.

**TABLE 4:** Content of the crisis of the first year.

Elements of the crisis	Indicators	Social situations
Intention for locomotion	Possibility to stand up and to make the first steps	Close presence of adult, who helps to realize the first steps
Intention for articulation of words	Possibilities to articulate syllables or simple words	Presence and affective communication of adult, who encourages pronunciation of the words together with the usage of objects
Intention for usage of cultural objects	Coordinate movements of both hands with the toys or objects	Presence of adult and presence of objects in practical situations of day-to-day life
Search for self-independence	Gestures, face expressions, crying with manifestation of necessity to be included into usage of new objects	Presence of adults who show interest in the child and approvement of his or her intentions

## Discussion and Conclusion

Psychological and neuropsychological literature commonly presents psychological development as synonymous to physical or physiological maturation, while psychological assessment of a child’s development is commonly reduced to assessment of movements, postures, and reflexes as progressive automatic changes during the first year (Gesell and Amatruda, 1980; Katona, 1988; Vojta, 2005; Bayley, 2015). In some proposals for assessment, mental development is reduced to natural progress in psychomotricity (Bayley, 2015). Frequently, different alterations of development are understood only as a result of brain damage or deficit of maturation, that is, are exposed as proper inner difficulties of the child and not as a result of the absence of joint activity between adult and child (Pelayo et al., 2016).

Vigotsky (1984) and his followers (Leontiev, 2000, 2003, 2009) have proposed another way of thinking. The child with or without difficulties is always a social child and is always a member of a social community. At the same time, psychology, in very few occasions, studies, what this community actually does with the child and how it guarantees psychological development. In this point our position, as positions of Vigotsky’s followers, is radical: an adult’s actions toward the child are the source of psychological development, but also the origin of the child’s stagnation. The term of interaction is not enough for understanding the causes of development. Social interactions are everywhere in society. On the contrary, the term of activity is a precise term for what the adult does and what the child does. Interaction is a generic general word, while activity is a precise concrete term, which is useful for psychological research.

Some recent studies have shown the importance of development of actions with cultural objects during the following psychological age. In many cases, speech disturbances are related to the absence of the variety of proper cultural actions with objects. At the same time, gradual introduction and development of the possibility of usage of objects and toys serve as the basis for positive appearance of verbal expression and understanding in children with developmental difficulties (Borges, 2020; Borges et al., 2020a,b). Such importance helps us to consider in an original way the possibilities of the influence and correction of development in cases of difficulties or risks.

The concept of social situation of development, as proposed Vigotsky (1984), helps us to understand the guiding role of organized activity and of orientation for communication and manipulation during the first year of life. In posterior periods of development, methodological relation between assessment and correction of difficulties should take into account the social situation of development (Solovieva and Quintanar, 2020). Organization of psychological development should always lead to psychological development (Solovieva and Quintanar, 2014, 2015; Veraksa and Sheridan, 2018).

The article has presented the content of the first crisis as the result of gradual psychological development during the first psychological age. The crisis of the first year includes the appearance of the first words or intention for pronunciation of the words, which should be understood differently from initial communicative intention, which manifests in the animation complex of the first three months. In terms of Bozhovich (1981, p. 132), the central new psychological formation of the first year is “affective representations” or “motivated representations.” We may refer to it as affective and motivated representation of intention for communication. Another element of the content of the first year is aspiration for independent movement (walking) toward objects, including the adult. The third and central element of the crisis is appearance of cultural action with objects (spoon as the prototype). The actions of the child are not precise yet, but the intention of their action is very clear. The whole complex of their own intentions for words, walking, and actions with objects might be assumed as the major independence of the child. The child expresses unconscious necessity of usage of cultural objects independently. At the same time, the communicative goals of the child are conscious, he or she may already ask for external help and express their own need with gestures, movements, expressions, and importantly, with their own independent words, which may have autonomous phonetic structure and autonomous meaning (Vigotsky, 1982, 1984).

During the period of the crisis, the child is ready to change the social situation of development and the guiding activity through the acquisition of proper cultural actions with proper cultural meaning, mediatized by oral language as “psychological mean by excellency” (Vigotsky, 1982). Understanding the content of the first psychological age and the indicators of the crisis would serve as the basis for proper clinical and psychological assessment and for organization of the measures of corrections. The content of new psychological formations and their early or late appearance in each psychological age should become the objects of profound psychological and neuropsychological studies. “Probably, in [the] nearest future, neuropsychological exploration of cortical functions in newborn child[ren] and in early age would permit not only to precise deviations, but also to know how to eliminate them in correspondent time and how to provide individual programs for optimal development of the child” (Skvortsov, 1995, p. 93).

## Data Availability Statement

The original contributions presented in the study are included in the article/supplementary material, further inquiries can be directed to the corresponding author.

## Ethics Statement

Written informed consent was obtained from the minor(s)’ legal guardian/next of kin for the publication of any potentially identifiable images or data included in this article.

## Author Contributions

The article is the result of the author’s research and practice, dedicated to developmental psychology according to cultural historical conception of child’s development. Both authors contributed to the article and approved the submitted version.

## Conflict of Interest

The authors declare that the research was conducted in the absence of any commercial or financial relationships that could be construed as a potential conflict of interest.

## Publisher’s Note

All claims expressed in this article are solely those of the authors and do not necessarily represent those of their affiliated organizations, or those of the publisher, the editors and the reviewers. Any product that may be evaluated in this article, or claim that may be made by its manufacturer, is not guaranteed or endorsed by the publisher.

## Footnotes

1 Photograph from the authors’ family archive.
